# Computed Tomography-based evaluation of porcine cardiac dimensions to assist in pre-study planning and optimized model selection for pre-clinical research

**DOI:** 10.1038/s41598-020-63044-1

**Published:** 2020-04-07

**Authors:** Miriam Lipiski, Matthias Eberhard, Thea Fleischmann, Sascha Halvachizadeh, Beate Kolb, Francesco Maisano, Mareike Sauer, Volkmar Falk, Maximilian Y. Emmert, Hatem Alkadhi, Nikola Cesarovic

**Affiliations:** 1 Division of Surgical Research, University Hospital Zurich, University of Zurich, Zurich, Switzerland; 2 Institute for Diagnostic and Interventional Radiology, University Hospital Zurich, Zurich, Switzerland; 3 Department of Trauma, University Hospital Zurich, Zurich, Switzerland; 4 Department of Cardiac Surgery, University Heart Center Zurich, Zurich, Switzerland; 5 Department of Cardiovascular Surgery, Charité Universitätsmedizin Berlin, Berlin, Germany; 6 Department of Cardiothoracic and Vascular Surgery, German Heart Institute Berlin, Berlin, Germany; 7 Department of Health Sciences and Technology, ETH Zurich, Zurich, Switzerland

**Keywords:** Cardiac device therapy, Preclinical research, Translational research

## Abstract

The pig (*Sus Scrofa Domestica*) is an accepted model for preclinical evaluation of prosthetic heart valves and trans-catheter implantation techniques. Understanding porcine cardiac dimensions through three-dimensional computed tomography (CT), increases preclinical study success, leading to higher cost efficiency and to the observance of the obligation to the 3 R principles. Cardiac CT images of twenty-four Swiss large white pigs were segmented; aortic root, mitral valve, pulmonary trunk, tricuspid valve, as well as the aorto-mitral angle and left atrial height were analyzed. Correlation coefficient (r) was calculated in relation to body weight. In Swiss large white pigs, valvular dimensions, length of the pulmonary artery and ascending aorta as well as left atrial height correlate with body weight. Coronary ostia heights and aorto-mitral angle size can be neglected in animal size selection; no changes were found for either of the two parameters with increasing body weight.

## Introduction

Driven by advancing technologies and skills, cardiology and cardiac surgery had been one of the medical fields quick to embrace minimally invasive techniques such as trans-catheter valve therapies in the early 2000s^1,2^. Minimally invasive techniques are generally accompanied by an improved patient satisfaction, a higher return to normality, a lesser requirement for post-rehabilitation services, and a marked reduction of costs^3^. Trans-catheter approaches have now been fully integrated into the therapeutic portfolio for managing patients suffering from cardiac disease with a risk too high for conventional surgery^4^, thus, leading to a high demand in development of new trans-catheter devices and new types of delivery systems^5^.

The use of large animal models in pre-clinical studies plays an important role in the initial evaluation of efficacy and safety of new medical devices before their use in human clinical trials^6–8^. The domestic pig (*Sus Scrofa Domestica*) is a widely accepted animal model of cardiovascular research and in particular for the preclinical evaluation of prosthetic heart valves and trans-catheter implantation techniques^9^. However, trans-catheter valve implantations in pigs, are often associated with the same complications as seen in humans, namely post-implantation para-valvular regurgitation, coronary occlusion and rupture of the aortic root or annulus due to oversizing of the valve^10,11^. Furthermore, left atrial volume and dimensions can be smaller in pigs for equivalent annulus size seen in humans^12^, therefore high profile valves in pigs may lead to obstruction or reduction of the pulmonary vein inflow and in case of touching the left atrial roof causing complications such as tissue erosion or cardiac arrhythmias^13^. High profile valves may also cause obstruction of the left ventricular outflow tract due to the geometric relationship between the aortic and the mitral valve^13^. Pre-procedural planning with modern imaging modalities such as trans-esophageal echocardiography and three-dimensional computed tomography (CT) have shown to lead to a better understanding of annular sizing and geometry and a reduction of these complications and have therefore become an integral part in the clinical assessment of patients^10,14–16^.

Unlike in the human patient, where the valve is chosen based on the patient’s annular size and geometry, in preclinical research a suitable animal is generally selected based on body weight for one particular valve size available for testing. Thus, the suitable animal must be large enough to accommodate human-grade devices, with the determining factors being the size of the target cardiac structure, as well as the diameter of the peripheral vessels used for vascular access^17^.

Studies in humans described a strong correlation between body surface area to normal aortic and pulmonary valve diameter. Other strong predictors of valve diameter include the patient’s age and body height^18^. Body weight and Body Mass Index (BMI) on the other hand are rather poor indicators of valve size in humans^18^. As for the pig, Allan *et al*. could also find a positive correlation between the body length and the aortic annulus and root diameter in the miniature swine^19^. Although miniature swine have become increasingly popular in research, the purchase price is markedly higher than for commercially raised farm breeds, which makes the latter more popular for acute and short term studies^20^. Additionally, most of miniature swine breeds will not reach the heart and blood vessel size which most closely approximates that of humans, precluding them from testing human-size implants^21^.

Implementing CT as an integral part of animal selection would be ideal however, although non-invasive imaging procedures are of low severity grade based on the severity classification of the EU Directive 2010/63^22^, they do require adequate sedation or general anesthesia and therefore an additional authorization from the competent authorities.

Therefore, in this study, cardiac CT images of commercial farm pigs of the same breed but with different body weights were used to perform morphometric analyses of porcine cardiac structures. The goal was to assess correlation between the pig’s body weight and the (intra) cardiac dimensions with the ultimate goal to allow for an accurate prediction of which animal would be the best weight-matched to test a particular size of cardiac implant. Thus, leading to an improved animal selection and enhanced preclinical study success.

## Methods

### Animal Study

Twenty-four domestic pigs (Swiss large white, intact females and castrated males) that had undergone pre-operative cardiac CT for a variety of different projects approved by the local Committee for Experimental Animal Research (Cantonal Veterinary Office Zurich, Switzerland) under the License numbers 152/2013, 219/2016 and 138/2017 were included into this study. Animal housing and all experimental procedures were in accordance with Swiss animal welfare protection law, and conform to European Directive 2010/63/EU of the European Parliament and the Council on the Protection of Animals used for Scientific Purposes, and to the Guide for the Care and Use of Laboratory Animals^23^.

Images were selected randomly and animals assigned to one of the following weight groups: 50–60 kg (n = 8), 80–90 kg (n = 8), 100–110 (n = 8).

### Animal Preparation

All pigs were sedated with an intramuscular injection of ketamine (Ketasol®-100 ad us.vet.; Dr. E. Graeub AG, Berne, Switzerland; 15 mg/kg body weight), azaperone (Stresnil® ad us.vet.; Elanco Tiergesundheit AG, Basel, Switzerland; 2 mg/kg body weight) and atropine (Atropinsulfat KA vet 0.1%; Kantonsapotheke, Switzerland; 0.05 mg/kg body weight). Anesthesia was induced by an intravenous administration of propofol (Propofol ®- Lipuro 1%, B. Braun Medical AG; Sempach, Switzerland; 1-2 mg/kg body weight) to achieve relaxation and swallow-reflex diminishment sufficient for intubation. Anesthesia was maintained during the duration of the study with propofol (5-10 mg/kg/h). For pain medication buprenorphine (Temgesic®; Indivior Schweiz AG, Baar, Switzerland; 0.01 mg/kg) was administered.

Animals were equipped with a 5 F sheath (Cordis AVANTI® + Introducer; Cardinal Health, USA) in the femoral vein or an 18 G auricular vein catheter (B. Braun Medical AG; Sempach, Switzerland) for contrast agent injection.

### CT imaging protocol and data reconstruction

Animals were scanned under general anesthesia with a third-generation 192-slice dual-source CT machine (SOMATOM Definition Flash, Siemens Healthineers, Forchheim, Germany). The data acquisition was synchronized with the electrocardiogram (ECG) of the animals using retrospective ECG-gating and using the following scan parameters: detector collimation 2 × 0.6 × 96, slice acquisition 2 × 0.6 × 192 by means of a z-flying focal spot, gantry rotation time 0.25 s, tube current–time product 380 mAs/rotation, and tube voltage 120 kVp. A total of 0.5–1 ml/kg BW iodinated contrast media (iopromide, Ultravist 370, Bayer Healthcare, Berlin, Germany) was administered intravenously at a flow-rate of 5 ml/s followed by 30 ml of saline chaser at a flow-rate of 3.5 ml/s.. Bolus tracking was performed in the ascending aorta. The contrast-enhanced CT scan was initiated after an attenuation threshold of 120 Hounsfield units [HU] at 100 kV was reached. Mean attenuation of the ascending aorta, left ventricle and right ventricle was 375 ± 130 HU, 368 ± 124 HU, and 367 ± 156 HU, respectively.

All CT data was reconstructed using a slice thickness of 0.6 mm and increment of 0.4 mm using advanced modeled iterative reconstructions (ADMIRE) at a strength level of 4. The reconstruction field-of-view (FoV) was set to 200 mm with a pixel matrix of 512 × 512. Images were reconstructed in 10% steps of the RR-interval.

### Data Analysis

**Figure 1:**
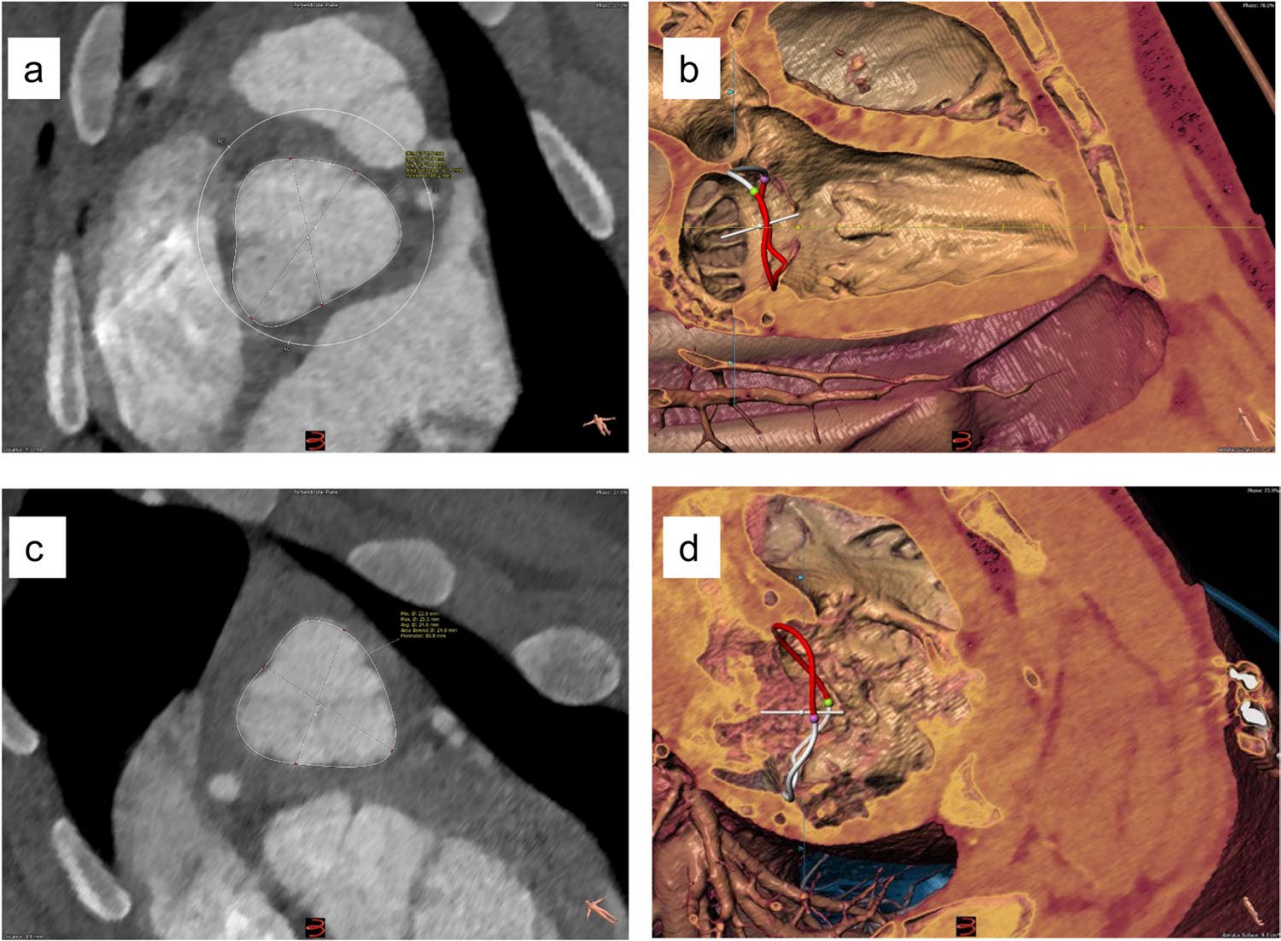
Representative Segmentation examples: Aortic Valve (**a**), Mitral Valve (**b**), Pulmonary Valve (**c**) and Tricuspid Valve (**d**).

**Figure 2:**
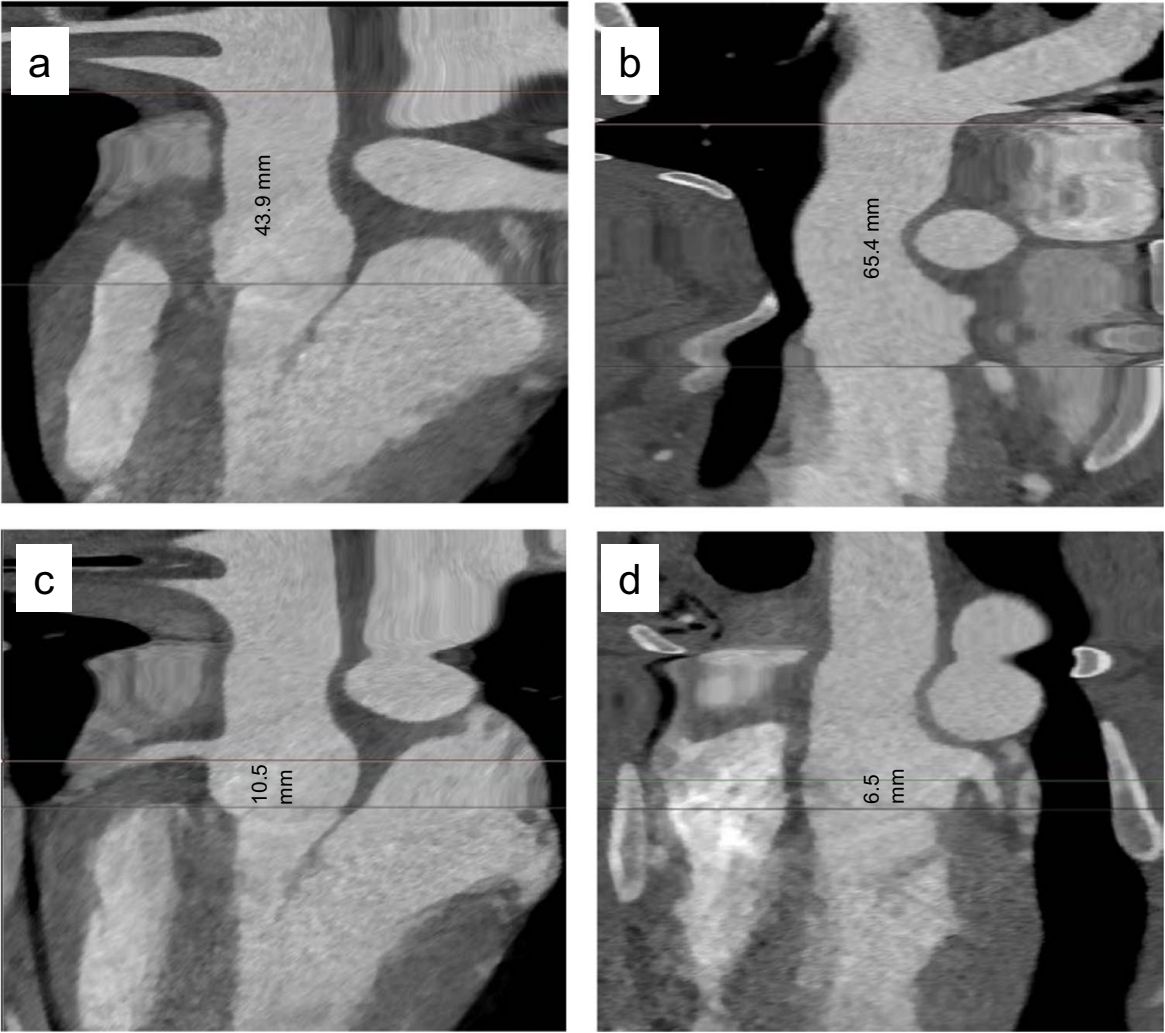
Representative Segmentation example: Length of ascending aorta (**a**), length of pulmonary trunk to bifurcation (**b**), RCA Ostium height (**c**) and LCA Ostium height (**d**).

All measurements were performed using 3mensio structural heart software Version 9.1 (3mensio Medical Imaging BV; Bilthoven; the Netherlands) (Figs. 1 and 2).

#### Aortic Root

Aortic root measurements were performed in diastole, following manual segmentation of the ascending aorta. The following measurements were acquired: diameter by area derived and perimeter derived annulus size; size of the left ventricular outflow tract (LVOT; 5 mm below the annulus plane). Length measurements were performed on the stretched vessel view, namely, length from annulus to the left coronary artery (LCA) ostia and the right coronary artery (RCA) ostia; length of the ascending aorta from annulus to the branch-off of the brachiocephalic trunk.

#### Mitral Valve

The following annulus dimensions were acquired in systole: Area; perimeter - in particular posterior perimeter, trigone-to-trigone (T-T) distance, septo-lateral (S-L) and commissure-to-commissure (C-C) distance.

#### Aortic-Mitral Angle

Aortic-Mitral Angle was measured in systole.

#### Pulmonary Trunk

Pulmonary trunk measurements were performed in diastole, following manual segmentation of the pulmonary artery. The following measurements were acquired: diameter by area derived and perimeter derived pulmonary annulus size; area and diameter of the pulmonary artery (20 mm above the annulus plane). Length measurements were performed on the stretched vessel view, namely, length from annulus to the bifurcation.

#### Tricuspid Valve

The following annulus dimensions were acquired in systole: area, perimeter, septo-lateral (S-L) distance, maximum diameter.

#### Left atrial height

left atrial height was measured in both systole and diastole.

### Statistical analyses

All analyses were performed with GraphPad Prism software version 8.0.0. Correlation coefficients (r) were computed using Spearman nonparametric correlation. Strength of association was defined as very weak correlation (positive and negative 0.01 to 0.19), weak correlation (positive and negative 0.20 to 0.39), moderate correlation (positive and negative 0.40 to 0.69), strong correlation (positive and negative 0.70 to 0.89) and very strong correlation (positive and negative 0.90 to 1.00). A two-tailed P value was computed, significance for all statistical tests was established at p ≤ 0.05. Correlation was determined between each column versus body weight. Columns assed were: all measurements acquired under aortic root, pulmonary trunk, mitral valve, tricuspid valve and left atrial height.

Additionally, a correlation matrix was created between all valve area sizes, LCA and RCA ostia height, coronary heights and length of ascending aorta.

## Results

### Aortic Root

**Figure 3:**
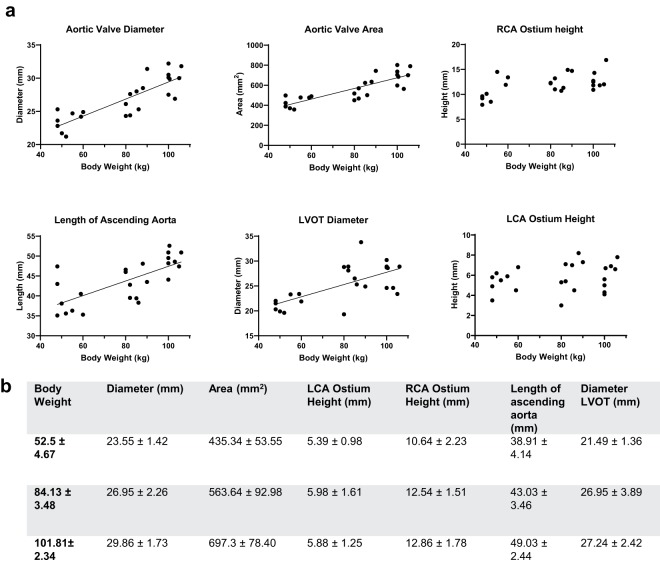
Aortic Root; a. Aortic Valve Diameter, Aortic Valve Area, RCA/LCA Ostium height, Length of Ascending Aorta, LVOT Diameter: Each parameter is correlated to body weight; each dot represents a single value. A linear regression line was fitted for parameters with moderate to strong correlation. (**b**) Values for the measured parameters are shown as Mean ± SD; Values are grouped in three groups of different body weights (50–60 kg (n = 8), 80–90 kg (n = 8), 100–110 (n = 8)).

A strong correlation was found between body weight and aortic valve diameter (r = 0.83, p < 0.0001) and aortic valve area, respectively (r = 0.84, p < 0.0001), as well as between body weight and length of the ascending aorta (r = 0.72, p < 0.0001) (Fig. 3). A moderate correlation was found between the body weight and LVOT diameter (r = 0.64, p = 0.0007). Correlation between body weight and LCA ostium was not significant (r = 0.32, p = 0.123), while the same correlation with the RCA ostium height was moderate (r = 0.52, p = 0.0095). Correlation between RCA ostium height and length of ascending aorta (r = 0.38, p = 0.066; r = 0.32) was not significant, similar to the correlation between LCA ostium height and length of the ascending aorta (r = 0.07, p = 0.757). Correlation between RCA and LCA ostia height was found to be moderate (r = 0.50, p = 0.014).

### Aortic-Mitral Angle

There was no significant correlation between body weight and aortic-mitral angle (r = 0.09, p = 0.66).

### Mitral Valve

**Figure 4:**
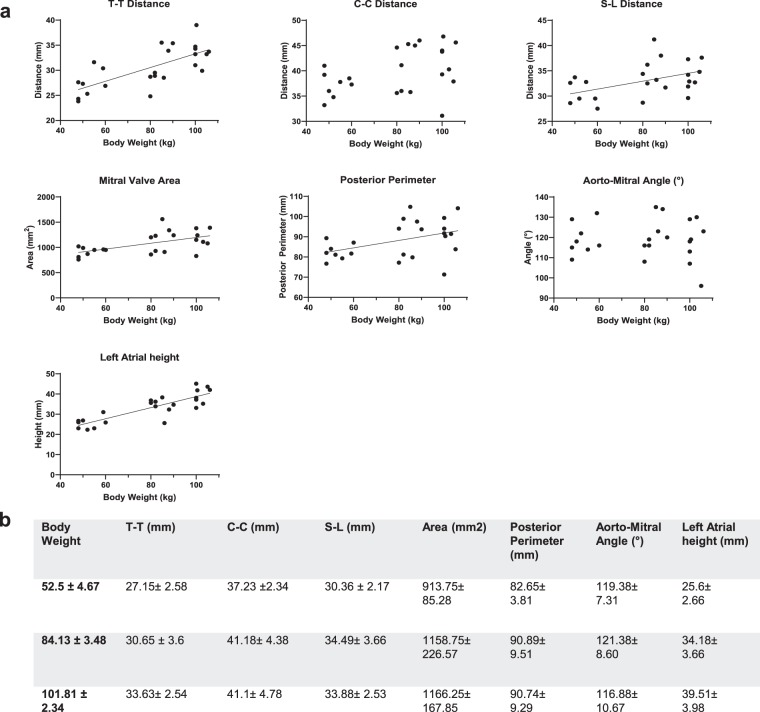
Mitral Valve; a. T-T Distance, C-C Distance, S-L Distance, Mitral Valve Area, Posterior Perimeter, Aorto-Mitral Angle and Left Atrial Height: Each parameter is correlated to body weight; each dot represents a single value. A linear regression line was fitted for parameters with moderate to strong correlation. (**b**) Values for the measured parameters are shown as Mean ± SD; Values are grouped in three groups of different body weights (50–60 kg (n = 8), 80–90 kg (n = 8), 100–110 (n = 8)).

A strong correlation was found between body weight and T-T distance (r = 0.73, p < 0.0001) as well as between body weight and left atrial height (r = 0.76, p < 0.0001) (Fig. 4). Correlations between body weight and mitral valve area, body weight and C-C distance, body weight and S-L distance as well as body weight to posterior perimeter was moderate (r = 0.56, p = 0.0037; r = 0.44, p = 0.0297; r = 0.49, p = 0.0143; r = 0.43, p = 0.0338).

### Pulmonary Trunk

**Figure 5:**
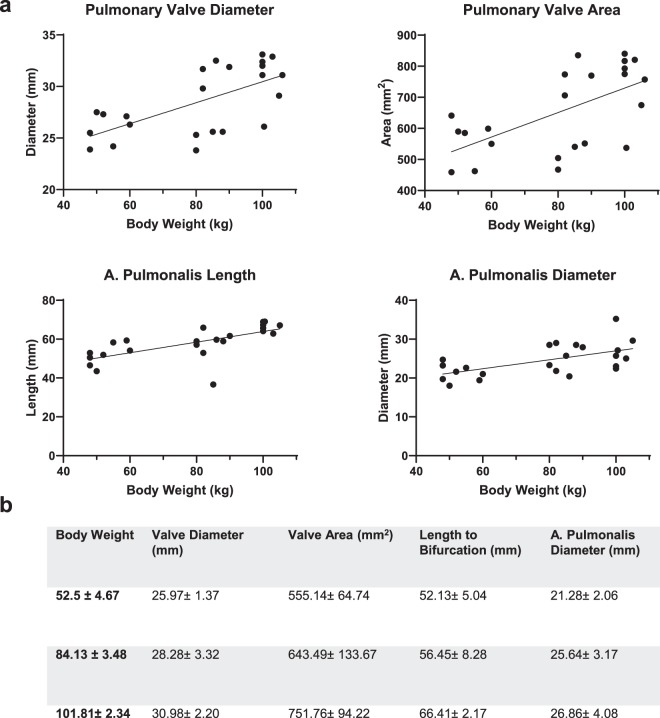
Pulmonary Trunk; (**a**) Pulmonary Valve Diameter, Pulmonary Valve Area, A.Pulmonalis Diameter, Length of A. Pulmonalis: Each parameter is correlated to body weight; each dot represents a single value. A linear regression line was fitted for parameters with moderate to strong correlation. (**b**) Values for the measured parameters are shown as Mean ± SD; Values are grouped in three groups of different body weights (50–60 kg (n = 8), 80–90 kg (n = 8), 100–110 (n = 8)).

A strong correlation was found between body weight and length of the pulmonary artery to bifurcation (r = 0.81, p < 0.0001) (Fig. 5). Correlations between body weight and pulmonary artery diameter (r = 0.55, p = 0.0059), pulmonary valve diameter (r = 0.61, p = 0.0019), and pulmonary valve area (r = 0.56, p = 0.0054) were moderate.

### Tricuspid Valve

**Figure 6:**
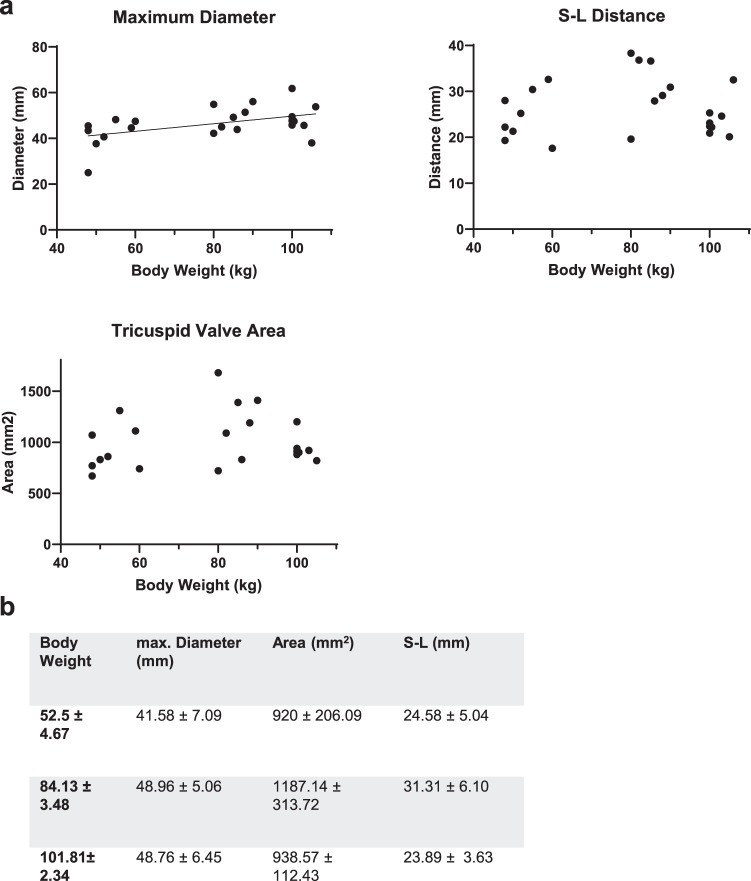
Tricuspid Valve; (**a**) Maximum Valve Diameter, S-L Distance, Tricuspid Valve Area: Each parameter is correlated to body weight; each dot represents a single value. A linear regression line was fitted for parameters with moderate to strong correlation. (**b**) Values for the measured parameters are shown as Mean ± SD; Values are grouped in three groups of different body weights (50–60 kg (n = 8), 80-90 kg (n = 8), 100–110 (n = 8)).

A moderate correlation was found between body weight and maximum tricuspid valve diameter (r = 0.46, p = 0.027) (Fig. 6). There was no significant correlation between body weight and tricuspid valve area, and between body weight S-L distance (r = 0.19, p = 0.3913; r = 0.04, p = 0.8520).

### Valve area sizes

Correlation matrix for all valve areas revealed the following correlations: A strong positive correlation was found between aortic valve area and mitral valve area (r = 0.70, p < 0.0001), correlation between aortic valve area and pulmonary valve area was moderate (r = 0.51, p = 0.014). A moderate correlation was also found between mitral valve area and tricuspid valve area (r = 0.59, p = 0.004). No significant correlation was found between aortic valve area and tricuspid valve area (r = 0.38, p = 0.081), between mitral valve area and pulmonary valve area (r = 0.15, p = 0.498), and between tricuspid valve area and pulmonary valve area (r = −0.12, p = 0.617).

## Discussion

Heart valve prosthesis are designated as high-risk medical devices, requiring rigorous *in-vitro* and *in-vivo* safety, efficacy and/or performance studies^24^. The use of standardized animal models for preclinical testing is thereby essential to provide invaluable information on the device safety. Choosing a suitable animal model for a particular experiment is of great importance and can significantly affect the outcome of a study^25,26^. Over the last decades, public concern about animal welfare in biomedical research has evolved markedly resulting in the 3R principles (i.e. replace, reduce, refine) being established worldwide as the ethical approach in regulating the use of animals for scientific purposes^27^. The aim of the refine-principle requires the use of improved experimental techniques particularly when testing new medical devices and biomaterials in animal models.

The pig has been widely used as a model in cardiovascular research^9^ with a broad acceptance in the literature that the anatomy of the pig’s heart is nearly identical to that of man^28–30^. In fact, trans-catheter valve implantations in pigs used for pre-clinical studies are often associated with the same complications as seen in humans^10,11^. However, unlike in humans, complications in pigs are often a consequence of a size mismatch, as preclinical studies are mostly performed with prototype devices of limited size variations^31,32^. While undersizing of prosthetic valves leads to para-valvular leakage and dislodgment of the prosthesis^33^ oversizing significantly impairs hemodynamic performance^34^. As both scenarios have a negative impact on the informative value of the preclinical study results, appropriate annular measurements and prosthesis sizing are considered critical. A better understanding of cardiac morphometries, including the inter-species differences and intra-species anatomical variabilities, is thereby essential to improve safety and efficacy in non-clinical device evaluation^14–16^ and to concurrently reduce the possibility of misleading and meaningless study results lacking transferability^35^.

Adequate pre-procedural planning with modern imaging modalities such as three-dimensional CT is successfully used in clinical patients to ensure appropriate prosthesis-patient size matching and should therefore also be used for the pre-selection of study animals. In previous publications, detailed analysis of porcine cardiac anatomy was mainly achieved by gross examination and dissection^12,36^. However, a study in human patients has shown, that measurements acquired by CT are significantly more precise than intraoperative direct measurements. *In-situ* measurements appear to be highly influenced by the unphysiological state the cardiac structures are assessed in as well as numerous structural conditions including angle of the great heart vessels and compliance of the valvular annulus^37^.

Three-dimensional evaluation of coronary ostia height by CT is especially fundamental when planning transcatheter aortic valve implantation (TAVI). In human patients, ostia heights greater than 12 mm from the annulus are commonly considered safe for TAVI procedures^38^. This study however, not only showed the left coronary ostia to be markedly lower in pigs than in humans (5.75 ± 1.33 mm vs. 13.4 ± 2.1 mm), but no increase of coronary ostia height could be found with advanced body weight of the animals. Low coronary ostia are associated with a higher risk for post-implant coronary obstruction, depending on the type of implanted prosthesis, resulting in myocardial ischemia and infarction^39^. The question whether the lower coronary ostia height in pigs makes them unsuitable in the testing of TAVI procedures or enhances the development of novel TAVI strategies for intermediate-risk patients (coronary ostia height <7 mm), remains open.

Transcatheter mitral valve replacement (TMVR) is mainly challenged by high-profile delivery systems accommodating large valve prosthesis, having to overcome an extreme angle within a relatively small space, when approaching transseptally via the atrium to reach the mitral valve^40^. Left atrial height in pigs has shown to be strongly correlating with pig’s body weight in this study. However, in pigs, the orientation of the heart along the cranio-caudal axes of the body resulting in a non-human cavo-apical angle poses an additional challenge on delivery devices^41^. Therefore, the transapical approach is a common alternative in TMVR procedures in pigs, making left ventricular dimension important factors to be considered in pre-surgical planning. Ventricular length shows no significant change with increased body weight in the animals included in this study (data not shown). Heart weight in modern farm pigs is thought to scale proportionally with body weight until the animals reach sexual maturity at the age of 4-5 months in males and 5-6 months in females^42^, corresponding to an approximate body weight of 80-90 kg. The relative heart weight later decreases as the animals continue to grow^36^; leading to the assumption that measurements determining size of intra-cardiac structures reflect the above-mentioned flattening of the curve with increased body weight. Previous studies have shown a positive correlation between the body length and the aortic annulus and root diameter in the miniature swine^19^. In the presented study a positive correlation between body weight and aortic valve diameter, aortic valve area and the length of the ascending aorta could be confirmed in Swiss large white pigs. Furthermore, a strong positive correlation between body weight and the length of the pulmonary artery to bifurcation was observed. However, most of the intra-cardiac dimensions assessed in this study appear to fail in complying or only moderately comply with natural scaling laws.

A few hypothesis for this non-compliance can be proposed: Firstly, in commercially bred farm pigs the rate of fat deposition increases during the growth stages, leading to an inaccurate reflection of body weight^43^. Prediction equations for estimating lean body mass in farm pigs are available, however, they are not well established and were therefore not applied in the presented study^44^. Secondly, deviations in measurements due to selected cardiac phases. As the software used for analysis was unable to perform the automatic segmentation of the ascending aorta in pigs, all measurements of the aortic root were performed with the valve closed to facilitate accurate manual identification of the most basal attachment points of the three aortic valve cusps needed for quantitative assessment of the annulus plane^45^. All other valves were subsequently also measured in the closed position although intra-cardiac cycle variations are known to have a marked influence on cardiac dimensions and indices of cardiac functions in humans^46^ with differences of up to 15% expected when using different cardiac phases^47^. Best correlation has shown to be achieved when measurements are done in mid-systole in 25–35% of the heart cycle^48^. Data on the amount of cyclical changes on annular dimensions in pigs, including cross-sectional area, perimeter and subsequently derived diameters however is more conflicting^34^. A dynamic geometry, mainly affecting the maximal diameter but not the minimal diameter, throughout the cardiac cycle is described for the left ventricular outflow tract and the mitral valve in pigs^35,36^. Possible further deviations in the measurements might be caused by high variations in heart rate between the animals, causing the respective cardiac phases, namely best systole and best diastole, to be defined at different percentiles of the RR interval.

Due to its retrospective nature, a further flaw in this presented study was the application of the contrast. A biphasic contrast injection protocols (80% high flow, 20% low flow) allowing for improved contrast in the right heart, while keeping the image of the left heart structures high and diagnostic^49,50^ was applied for all recordings of porcine cardiac CT scans. However, as the contrast was given over either the auricular vein or the femoral vein, inhomogeneity of contrast in the right ventricle was greater in the animals with auricular vein access due to the larger amount of non-contrasted blood mixing in from the inferior vena cava. The resulting reduction in image quality of the right heart partially hampered the accurate reconstruction of the anatomy and dimensions of the tricuspid valve and the pulmonary valve respectively.

The most common breeds of domestic swine used in biomedical research found in the literature are Yorkshire, Landrace, Duroc, Pietrain and crossbreeds such as the Swiss Large White used in this study^51^. Landrace, Duroc, Pietrain and crossbreeds appear to have a similar linear growth curve^52,53^,while Yorkshire pigs are described to significantly differ by having a curvilinear growth pattern^54^. The breed difference in rate of growth of heart relative to total muscle mass has been found to be not significant in Pietrain and Large White Pigs^53^. Although the current study included castrated males and intact females of only Swiss Large White Pigs, potentially neglecting an influence of sex and breed, similar data in pigs of breeds with similar growth patterns might be speculated.

Last, pre-procedure planning for trans-catheter implantation studies requires thorough knowledge of cardiac dimensions to pick a suitable study animal size. However, vascular access route, including aorto-iliac vessel assessment (angulation, luminal size) and length and diameter of the abdominal aorta for delivery system length and diameter suitability is equally crucial for animal selection^48^. Further studies are needed to complete these missing parameters.

In conclusion, blindly correlating the body weight of a domestic swine breed, to all intra-cardiac structures leading to an accurate prediction of which pig would be the best size-matched to test a particular cardiac implant, appears unfeasible based on the acquired data. However, there are a few statements that can be made: Firstly, valvular diameters and area sizes moderately to strongly correlate with pig’s body weight of this particular swine breed and possibly of swine breeds with similar growth curve patterns. The here collected data can therefore serve as a future guide in prosthetic valve size selection. Secondly, coronary ostia height and aorto-mitral angle size can be neglected in animal size selection as no change was found for either of the two parameters with increasing body weight. Lastly, length of the pulmonary artery to bifurcation as well as length of the ascending aorta can be well correlated with body weight.
